# *Gata2a* Mutation Causes Progressive Microphthalmia and Blindness in Nile Tilapia

**DOI:** 10.3390/ijms24043567

**Published:** 2023-02-10

**Authors:** Xingyong Liu, Li Zhou, Wenbo Li, Jiahong Wu, Deshou Wang

**Affiliations:** Integrative Science Center of Germplasm Creation in Western China (CHONGQING) Science City, Key Laboratory of Freshwater Fish Reproduction and Development (Ministry of Education), Key Laboratory of Aquatic Science of Chongqing, School of Life Sciences, Southwest University, Chongqing 400715, China

**Keywords:** GATA2, lens fiber cell, microphthalmia, blindness

## Abstract

The normal development of lens fiber cells plays a critical role in lens morphogenesis and maintaining transparency. Factors involved in the development of lens fiber cells are largely unknown in vertebrates. In this study, we reported that GATA2 is essential for lens morphogenesis in Nile tilapia (*Oreochromis niloticus*). In this study, Gata2a was detected in the primary and secondary lens fiber cells, with the highest expression in primary fiber cells. *gata2a* homozygous mutants of tilapia were obtained using CRISPR/Cas9. Different from fetal lethality caused by *Gata2*/*gata2a* mutation in mice and zebrafish, some *gata2a* homozygous mutants of tilapia are viable, which provides a good model for studying the role of *gata2* in non-hematopoietic organs. Our data showed that *gata2a* mutation caused extensive degeneration and apoptosis of primary lens fiber cells. The mutants exhibited progressive microphthalmia and blindness in adulthood. Transcriptome analysis of the eyes showed that the expression levels of almost all genes encoding crystallin were significantly down-regulated, while the expression levels of genes involved in visual perception and metal ion binding were significantly up-regulated after *gata2a* mutation. Altogether, our findings indicate that *gata2a* is required for the survival of lens fiber cells and provide insights into transcriptional regulation underlying lens morphogenesis in teleost fish.

## 1. Introduction

Lens morphogenesis is a key event during eye organogenesis, and abnormal lens morphogenesis results in a range of lens/eyes structural abnormalities and cataract formation [1,2,3]. Genetic studies have revealed many genes implicated in microphthalmia in mammals [4]. These genes are involved in a variety of molecular pathways and include transcription factors *Pax6*, *Otx2*, *Sox2*, *Vsx2*, *Six3* and *Sall2* [5,6], crystallin genes (*Cryaa*/*b*, *Crybb1*/*2*/*3*, *Cryba1*/*2*/*3*/*4* and *Crygc*/*d*/*s*), membrane protein genes (*Gja3*/*8*, *Mip* and *Lim2*) [7,8], growth and transcription factor genes (*Pitx3*, *Maf* and *Hsf4*) [9] and beaded filament structural protein genes (*Bfsp1* And *Bfsp2*) [10]. In contrast to the more complexly shaped lens of mammals, the lens of fish presents a simpler structure. Although findings in mammals have made tremendous contributions to our understanding of the genetic etiology of microphthalmia, new causative genes as well as the underlying molecular mechanisms remain to be discovered in teleosts.

The lens is composed of a single cell type that follows a developmental pattern, beginning as a member of the germinative zone in the single layer of anterior epithelial cells overlaying the fiber cell mass [11,12]. The lens contains the anterior lens epithelium and the posterior lens fibers, which consist of the primary and secondary lens fiber cells [5,13,14]. Fiber cells make up the lens nucleus; both the ordered arrangement of the fiber cells and their sutures, as well as their intracellular structure, are important for light transmission and lens transparency [11,15,16,17]. To obtain transparent lens, both primary and secondary lens fiber cells undergo terminal differentiation including cell cycle exit, burst expression of crystallin proteins, removal of subcellular organelles, such as mitochondria, endoplasmic reticulum, Golgi apparatus and nuclei [18,19]. The degradation of lens fiber cell organelles produces the organelle free zone, and the defect of this process leads to cataract formation [12]. Studies in the development of lens fiber cells have been relatively clear in mammals, but little is known regarding teleosts.

GATA2 is a highly conserved transcription factor with a zinc-finger domain, and with a major role in hematopoietic, cardiovascular, neural and urogenital systems from Drosophila to humans [2,20]. GATA2 is expressed in numerous mammalian tissues, including hematopoietic, cardiovascular, neural and urogenital systems, and yet it plays important roles in the regulation of tissue-restricted gene expression [21,22,23]. In humans, mutations in GATA2 result in four clinical syndromes: monocytopenia, mycobacterial infections syndrome, B and NK lymphoid deficiency and acute myeloid leukemia [23,24,25,26]. Mice lacking GATA2 die at embryonic day (E) 10.5 with defects in primitive and definitive hematopoiesis [27]. Genome duplication within the teleost lineage has yielded two *Gata2* paralogons that have been maintained in these jawed fish, including zebrafish and tilapia [2]. Recent studies have revealed that the function of mammalian GATA2 is split between Gata2a and Gata2b in zebrafish [2,28]. Gata2a is required for hemogenic endothelium specification upstream of Gata2b. Gata2b is not vital for the embryonic generation of hematopoietic stems and progenitor cells, but supports their expansion in caudal hematopoietic tissue [28,29]. However, during adulthood, Gata2b is required for the quiescent hematopoietic stem and progenitor cell (HSPC) population [29]. A mutation of *gata2a* disrupts dorsal aorta morphogenesis and leads to embryo death. *gata2b*-deficient zebrafish have a reduction in embryonic definitive HSPC numbers, but are viable [28,29]. In addition to its critical role in hematopoiesis, due to the embryonic lethality of *gata2a* mutations, it is unclear whether it is involved in the regulation of the development of other tissues and organs.

Nile tilapia are the only farmed fish in the world capable of large-scale genetic manipulation and gene mutation. They have a high-quality genome sequence, a short spawning cycle (14 days at 26 °C), large spawning capacity and can reproduce all year round. They can provide sufficient material for studying the tissue and organ development of cultured fish. In the present study, homozygous mutants of *gata2a* and *gata2b* were constructed in Nile tilapia using the CRISPR/Cas9 system. A single mutation in *gata2a* or *gata2b* did not cause embryonic lethality. Interestingly, *gata2a* mutants displayed progressive microphthalmia and blindness. Expression profiling analysis showed that Nile tilapia Gata2a was highly expressed in primary lens fiber cells and weakly expressed in secondary lens fiber cells during lens morphogenesis. Loss of *gata2a* resulted in the death of primary lens fiber cells. Transcriptome profiling showed that the expression levels of genes coding crystallin were significantly down-regulated, while the expression levels of genes involved in visual perception and iron ion binding were significantly upregulated in the *gata2a* homozygous mutants. Altogether, our data revealed that Gata2a is essential for lens morphogenesis in Nile tilapia and provided insights into transcriptional regulation of lens morphogenesis in teleost fish.

## 2. Results

### 2.1. Expression Pattern of Gata2a in the Lens of Nile Tilapia

**Figure 1:**
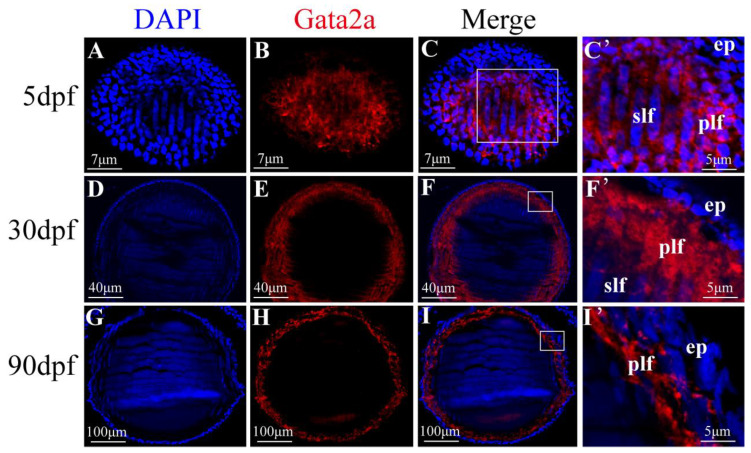
Expression pattern of Gata2a in the lens of Nile tilapia at different developmental stages. (**A**–**C’**) Expression of Gata2a was detected in the fiber cell mass of the lens from larvae at 5 dpf. (**D**–**F’**) Gata2a expression was detected in the nucleus of primary and secondary (anterior and posterior elongating) lens fiber cells, most prominently in the primary lens fiber cell near the equatorial zone where differentiation first initiates, from juvenile at 30 dpf. (**G**–**I’**) Gata2a was consistently detected in the nuclei of primary fiber cells along the equatorial zone of the lens at 90 dpf. (**C’**,**F’**) was the higher magnification fluorescent micrograph of the boxed region shown in (**C**,**F**), respectively. dpf: day post fertilization. ep: epithelial cell; plf: primary lens fiber cell; slf: secondary lens fiber cell.

According to the transcriptome data of Nile tilapia (*Oreochromis niloticus*) from NCBI (BioProject: PRJNA78915), *gata2a* was broadly expressed in the eyes, heart, brain and kidney, with the highest level in the eyes (Figure S1). To better understand the roles of Gata2a in eye development, we conducted immunofluorescence to profile dynamic spatial-temporal expression of Gata2a in Nile tilapia eyes. Expression of Gata2a was detected in the primary and secondary lens fiber cells (anterior-posterior elongation) from larvae at 5 days post fertilization (dpf), most prominently in the primary lens fiber cells (Figure 1A–C’). When secondary lens fiber cells began to elongate to form the concentric layers, Gata2a immunoreactivity was also detected in the nucleus of primary and secondary lens fiber cells from juveniles at 30 dpf, most prominently in the primary lens fiber cells (Figure 1D–F’). Gata2a immunoreactivity was consistently detected in the nuclei of primary and secondary lens fiber cells along the surface layers at 90 dpf (Figure 1G–I’).

### 2.2. Establishment of Gata2a and Gata2b Homozygous Mutants in Nile Tilapia

**Figure 2:**
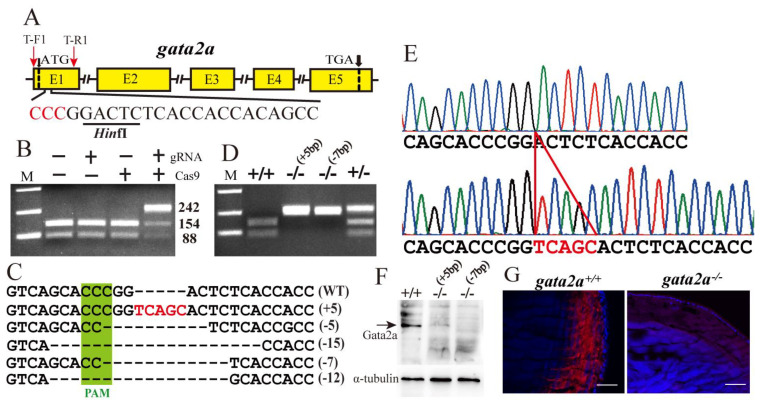
Generation of tilapia *gata2a* mutant line by CRISPR/Cas9. (**A**) Schematic diagram showing *gata2a* gene structure and sgRNA target site near the translation start site on the antisense strand. (**B**,**C**) Generation of the *gata2a* mutant line. Mutations at the *gata2a* locus were analyzed by restriction enzyme digestion (**B**) and DNA sequencing (**C**). Deletions are indicated by dashes (**C**). The numbers in brackets represent the bases deleted/inserted from each allele. (**D**) Identification of F2 genotypes by restriction enzyme digestion assay. Homozygous mutants with different mutation types (+5 bp and −7 bp) were obtained. (**E**) Sequencing results of the *gata2a* gene from WT and homozygous mutant fish. (**F**) Western blot was carried out to show the specificity of the antibody against Gata2a. (**G**) Immunofluorescence was used to test the specificity of the Gata2a antibody. Scale bar: 50 μm. M, marker; PAM, protospacer adjacent motif.

The *gata2a* and *gata2b* mutants were obtained using the CRISPR/Cas9 system in Nile tilapia. The genomic target sites were located in the region adjacent to the translational start site within the open reading frame of the *gata2a* and *gata2b* genes (Figure 2A and Figure S2A). The guide RNA (gRNA) and Cas9 mRNA were co-microinjected into single-cell embryos (fertilized eggs). Subsequent PCR using genomic DNA from eggs at 72 h after injection (time required for gRNA and Cas9 mRNA to be translated into protein and working) as a template, followed by restriction digestion (*Hin*fI), revealed three bands with an obvious uncut band in the PCR products of the *gata2a* mosaic mutants, but revealed two bands in the controls (Figure 2B and Figure S2B), indicating that the *gata2a* and *gata2b* gene had been mutated. Further sequencing analysis revealed that, compared with the WT (*gata2a*^+/+^ and *gata2b*^+/+^), the positive F0 mutants harbored different types of genomic insertion/deletion around the target sites of *gata2a* and *gata2b* ORF (Figure 2C and Figure S2B). The F1 mutants carrying 5-bp insertion and 7-bp deletion were used to establish *gata2a* homozygous mutants (Figure 2E,F and Figure S3A,B). The F1 mutants carrying 2-bp insertion were used to establish *gata2b* homozygous mutants (Figure S2). Genotyping analysis of the F2 generation showed that homozygous mutants carrying frameshift mutation in *gata2a* (5-bp insertion and 7-bp deletion) and *gata2b* (2-bp insertion) were successfully obtained (Figure 2D,E, Figures S2D,E and S3A–C). The *gata2a* and *gata2b* mRNA levels were significantly down-regulated in the mutants (Figures S2F and S3F). Western blot was carried out to show the specificity of the antibody against Gata2a (Figure 2F). Immunofluorescence assay confirmed that a specific signal of *gata2a* was detected in the lens of WT, while it was absent in the lens of *gata2a* homozygous mutants (Figure 2G).

### 2.3. Gata2a Mutation Causes Microphthalmia and Blindness in Nile Tilapia

**Figure 3:**
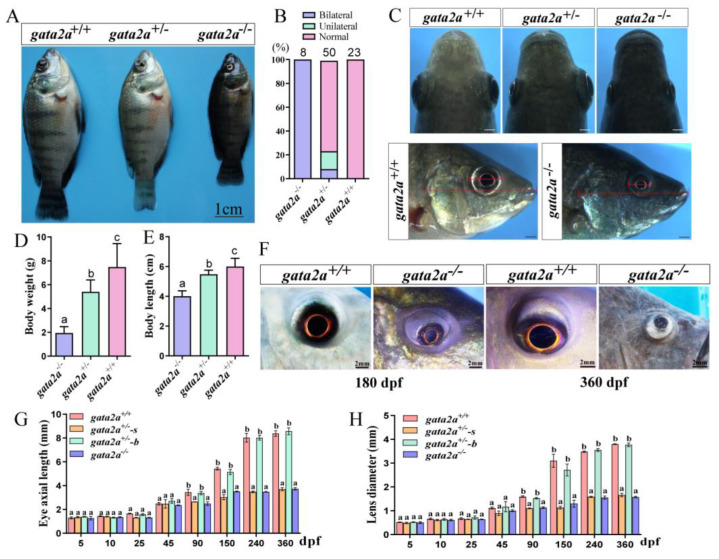
Morphological analysis of tilapia *gata2a* mutants at 90 dpf. (**A**) Morphology of *gata2a* mutants. (**B**) Proportions of the fish with microphthalmia in the F2 generation. (**C**) Eyes morphology of the *gata2a* mutants at 90 dpf. The presence of small eyes on the left or right side is random in *gata2a*^+/−^ fish. (**D**,**E**) Body weight and body length (sample size: homozygous mutants, *n* = 8, heterozygous mutants, *n* = 50, wt, *n* = 23). (**F**) Eye morphology of *gata2a* homozygous mutants at 180 and 360 dpf. (**G**,**H**) Eyes axial length and lens diameter. Data are expressed as the mean ± SD. One-way ANOVA was performed followed by Tukey’s test. Different letters above the error bar indicate significant differences at *p* < 0.05. dpf: day post fertilization. “*gata2a*^+/−^-s” and “*gata2a*^+/−^-b” represents eyes from *gata2a* heterozygous mutants with unilateral micropgthalmia (s, small eye; b, big eye).

During the development of the mutants, we found that some homozygous mutants of *gata2a* (4/10) and *gata2b* (6/10) showed abnormal hematogenesis (anemia of the heart, congestion of the head, eyes, or tail) and died after hatching (Figure S3D). The remaining mutants survived, and developed to the adult stage. Interestingly, we found some *gata2a* mutants with microphthalmia and darker body color at the later developmental stages (Figure 3A). Homozygous mutants with 5-bp insertion displayed similar defects as those with 7-bp deletion (Figure S3E). During subsequent analysis, homozygous mutants with 5-bp insertion were used. In the F2 generation, genotyping analysis indicated that all the *gata2a* homozygous mutants displayed microphthalmia (8/8), while only some of the heterozygous mutants displayed microphthalmia. Among the heterozygous mutants with microphthalmia, about 15/100 exhibited unilateral microphthalmia (8/50), while about 8/100 exhibited bilateral microphthalmia (4/50) (Figure 3B,C). The body weight and body length were significantly decreased in the homozygous mutants (Figure 3D,E). At 180 dpf, the lens of *gata2a* homozygous mutants were smaller and less transparent than that of WT. Lens degeneration was observed at 360 dpf, which led to blindness in adulthood (Figure 3F). At 5, 10, 25 and 45 dpf, there were no significant differences in axial length and lens diameter between mutants and WT (Figure 3G,H). In *gata2a* homozygous mutants, the eye axial length and lens diameter significantly decreased at 90, 150, 240 and 360 dpf. Consistently, the eye axial length and lens diameter from the small eyes (*gata2a*^+/−^-s) also significantly decreased at those developmental stages compared to that of big eyes (*gata2a*^+/−^-b) in the heterozygous mutants with unilateral microphthalmia (Figure 3G,H).

### 2.4. Gata2a Mutation Disrupts the Development of the Lens Fiber Cells

**Figure 4:**
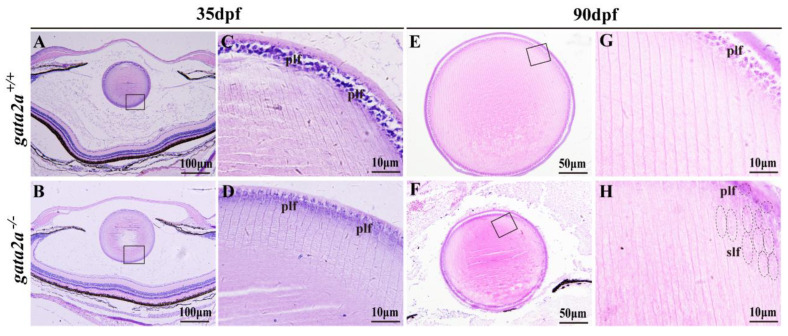
Histological analysis of the eyes from WT and *gata2a* mutants at 35 and 90 dpf. (**A**–**D**) Histology of the eyes at 35 dpf, the size of the lens from *gata2a* mutants were similar to that of WT (**A**,**B**), while most of the primary lens fiber cells were degenerated (**C**,**D**). (**E**–**H**) Lens from *gata2a* mutants were smaller and opaque compared to those from WT siblings (**E**,**F**). Most of the primary lens fiber cells in the *gata2a* mutants were degenerated, and the nuclei were not degraded in some of the secondary lens fiber cells (**G**,**H**). (**C**,**D**,**G**,**H**) are magnifications of the boxed areas in (**A**,**B**,**G**,**H**), respectively. dpf: day post fertilization. plf: primary lens fiber cell; slf: secondary lens fiber cell. The dotted circles and ellipses in the (**H**) diagram represent primary and secondary lens fiber cells, respectively.

Histological analysis of the lens at 35 dpf showed that most of the primary lens fiber cells in the gata2a mutants were degenerated compared with those of the WT (Figure 4A–D). At 90 dpf, compared with WT, gata2a mutants had a smaller lens and were opaque vitreous (Figure 4E,F). In the gata2a mutants, the primary lens fiber cells also largely degenerated, and compared with the WT, the fibers were discontinuous and disordered. Moreover, the degradation of fiber cell nuclei, a marker for terminal differentiation of secondary lens fiber cells, did not take place in some of the secondary lens fiber cells of the gata2a mutants. This starkly contrasted with the normal lens development in the WT siblings (Figure 4G,H).

### 2.5. Gata2a Mutation Down-Regulates Genes Related to Crystallin Synthesis and Up-Regulates Genes Related to Visual Perception and Iron Ion Binding

**Figure 5:**
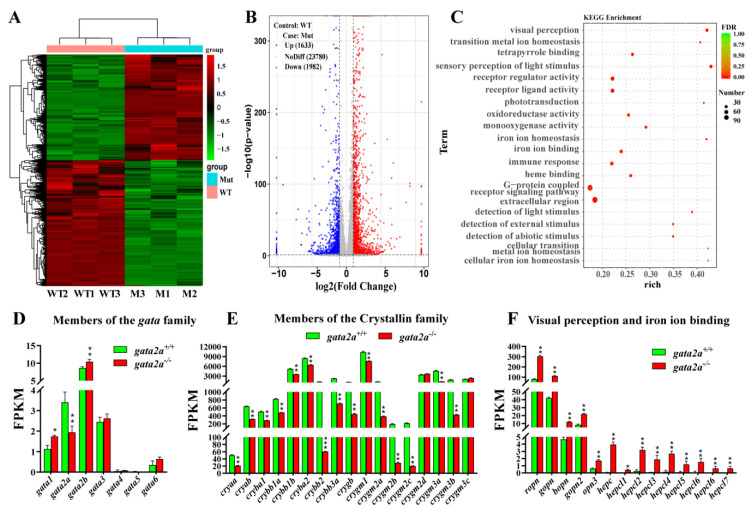
Comparative transcriptomic analysis of genes and molecular pathways dysregulated in *gata2a*^−/−^ eyes. (**A**) Heatmap displaying difference in gene expression between eyes from WT (*gata2a*^+/+^) and *gata2a* mutants (*gata2a*^−/−^) at 35 dpf. (**B**) Volcanic map of differentially expressed genes (DEGs) between the eyes from WT and *gata2a* mutants. A total of 1982 and 1633 genes were downregulated and upregulated, respectively, in the eyes from *gata2a* mutants compared with the eyes from WT fish. (**C**) Scatter plot of the enriched KEGG pathways for the DEGs. The sizes and colors of the dots represent the number of genes and the significance of the difference, respectively. (**D**) Effects of *gata2a* mutation on gene expression of the *gata* family. (**E**,**F**) *Gata2a* mutation down-regulated crystallin family genes (**E**), and up-regulated visual perception and iron ion binding-related genes (**F**) in the eyes. Data were presented as the mean ± SD of triplicates. Differences between groups were statistically examined with two-tailed unpaired Student’s *t*-test. Significant difference is denoted by * (*p* < 0.01), ** (*p* < 0.001) and *** (*p* < 0.0001).

To determine how Gata2a transcriptionally regulates lens morphogenesis in Nile tilapia, we conducted high-throughput RNA sequencing (RNA-Seq) analysis to assess *gata2a* mutation-induced changes in the eye transcriptome at 35 dpf. Comparative analysis showed that 1982 and 1633 genes were downregulated and upregulated in the eyes, respectively, after the *gata2a* mutation (Figure 5A,B). Kyoto Encyclopedia of Genes and Genomes (KEGG) enrichment analysis showed that these differentially expressed genes were mainly enriched in the pathways related to visual and sensory perception and iron ion homeostasis (Figure 5C). Expression of the members of the Gata family was analyzed, and we found that the expression level of *gata2a* was significantly down-regulated, while *gata1* and *gata2b* were up-regulated (Figure 5D). Notably, we found that the expression of the member of crystallin family genes, including the α- crystallin coding genes *cryaa* and *cryab*, the β-crystallin coding genes *cryba1*/*2*, *crybb1a*/*b*, *cryba2*, *crybb2* and *crybb3a*, and some of the γ-crystallin coding genes *crygm1*, *crygm2a*/*2b* and *crygm3a*/*b*, were significantly down-regulated in the eyes of *gata2a*^−/−^ fish (Figure 5E). In contrast, the opsin-related genes, including *ropn*, *gopn2*, *gopn3*, *bopn* and *opn3*, and members of the iron ion binding genes *hepc*, *hepcl1*/*2*/*3*/*4*/*5*/*6*/*7*, were significantly up-regulated in the eyes of *gata2a*^−/−^ fish (Figure 5F).

## 3. Discussion

In the present study, we demonstrated that Gata2a expression began in the fiber cell mass of the developing lens, and continued to be expressed in primary and secondary fiber cells throughout lens development. Consistent with its spatiotemporal expression in the developing lens, the absence of *gata2a* led to the death of primary fiber cells, resulting in progressive microphthalmia and blindness.

The GATA family of zinc finger domain transcription factors in vertebrates includes six members, GATA1-6, which all bind to a consensus ‘‘A/T-GATA-A/G’’ DNA motif [30]. GATA factors are important for the development of several organs controlling proliferation, differentiation and movement, as well as cell-fate specification [20]. Among the six GATA members, GATA2 is the most widely studied because of its important role in the hematopoietic system. Teleost fish have undergone a third whole genome duplication (3R) specific to their lineage, resulting in the retention of multiple gene paralogs. However, only *Gata2* of the six members retains two paralogons in teleosts [2]. In tongue sole (*Cynoglossus semilaevis*) and Japanese flounder (*Paralichthys olivaceus*), selection pressure analysis predicted that these gene duplicates experienced purification selection and possible neo-functionalization [2]. In this study, we found that *gata2a* plays an important role in lens morphogenesis, which has not been previously reported. A study in mice showed that another member of the GATA family, GATA3, is also essential for the development of the lens [31]. Phylogenetic analysis showed that Gata3 and Gata2a/b of teleosts were clustered into two independent branches with GATA3 and GATA2 of other vertebrates, respectively, indicating that GATA2 and GATA3 independently evolved (Figure S4) [2]. In the present study, *gata1* and *gata2b* were up-regulated in the *gata2a* homozygous mutants, while *gata3* was not. These results suggest that lens defects caused by *gata2a* mutation in Nile tilapia are not related to *gata3*. The up-regulation of *gata2b* expression in *gata2a* homozygous mutants suggests that *gata2b* may compensate for the absence of *gata2a*, but further investigations are needed to provide more evidence in future studies. Whether Gata3 also participates in the development of teleost lens remains to be studied.

Regulation of cell cycle by GATA factors has been reported in many different tissues. Studies have shown that neural epithelial cells of GATA2-deficient mouse embryos exhibit abnormal proliferation, and overexpression of GATA2 induces neural differentiation by inhibiting the proliferation of neural progenitor cells [32]. In erythroid cell differentiation, GATA1 has been reported to induce erythro-megakaryotic differentiation by suppressing the active cell cycle of hematopoietic progenitor cells [33]. GATA-3 has been reported to suppress the abnormal proliferation of mesonephric cells, as well as mammary epithelial cells [34]. Conditional deletion of GATA-3 in mice resulted in an abnormal cell cycle and the failure of lens fiber cell differentiation [31]. These reports and current observations suggest that the death of lens fiber cells caused by the *gata2a* mutation may be related to the abnormal cell cycle.

Crystallin proteins, which make up about 90% of the water-soluble protein, are highly concentrated and densely packed structural proteins of lens fiber cells [35,36,37]. The differentiation of lens fiber cells is also characterized by the expression and accumulation of crystallins. If these proteins aggregate, the light-scattering aggregate formed is called a cataract, the most common cause of blindness [35]. Of the crystallin subtypes, α-crystallins are normally expressed in both lens epithelial and fiber cells, and are also powerful inhibitors of lens fiber cell apoptosis [11,38,39]. In the *gata2a* homozygous mutants, most of the α-crystallins were down-regulated, which may lead to the apoptosis of lens fiber cells. During differentiation, mature lens fiber cells produce abundant β- and γ-crystallins [36]. β-crystallin expression, which begins at E11 in the mouse embryo, serves as an early marker of fiber cell differentiation [40,41,42]. We showed here that expressions of all the α- and β-crystallin coding genes were significantly down-regulated in the lens of *gata2a* homozygous mutants. These data indicated that disrupted lens fiber cell differentiation in the *gata2a* homozygous mutants may be attributed to the down-regulation of α- and β-crystallin gene expression. Changes in metal ions could also affect the extracellular matrix, and an accumulation of iron ion contributed to cataractogenesis [37,43]. In *gata2a* homozygous mutants, the expression of visual perception and iron ion binding-related genes was up-regulated, which may lead to the accumulation of metal ions in the lens, thus leading to the occurrence of cataracts and blindness.

In conclusion, we demonstrated here that Gata2a was specifically expressed in primary and secondary fiber cells of the lens, and played an essential role in normal lens morphogenesis. Loss of *gata2a* resulted in the death of primary lens fiber cells, and progressive microphthalmia and blindness. Further studies will be necessary to determine how Gata2a functionally coordinates cell cycle with normal development of the lens fiber cells. Given that *gata2a* mutation altered the expression of genes associated with crystallin synthesis and visual perception and iron ion binding, it will also be of interest to establish a link from these genes to lens morphogenesis in Nile tilapia. In addition, how *gata2a* regulates the transcription of its downstream targets remains to be further investigated.

## 4. Materials and Methods

### 4.1. Animals

The founder strain of Nile tilapia (*Oreochromis niloticus*) was obtained from Japan (introduced from Egypt in Africa, 1970s), and kept in recirculating freshwater tanks at 26 °C under natural photoperiod. Experimental fish were reared in glass tanks in recirculating aerated freshwater with approximately pH 7.5 at 26 °C under natural photoperiod conditions and appropriate rearing density, namely 100 fish (10 dpf), 30 fish (60 dpf), 20 fish (90 dpf) and 7 fish (180 dpf) in 60 L fish tanks. Three adult normal females (XX) and three normal males (XY) (mean body weight 0.5 kg, mean total length 28 cm) were mated to obtain the fertilized eggs for injection. The use of animals was in accordance with the regulations of the ethics committee of Southwest University (No. IACUC-20181015-12, 15 October 2018).

### 4.2. Construction of Gata2a and Gata2b Homozygous Mutants by CRISPR/Cas9

The sequences of the Nile tilapia *gata2a* (Gene ID: 100705840) and *gata2b* gene (Gene ID: 100697130) were obtained from NCBI. The Nile tilapia *gata2a* mutants were generated with the CRISPR/Cas9 approach as previously described [44]. The gRNA (250 ng/μL) and Cas9 mRNA (500 ng/μL) were co-injected into one-cell stage embryos with a microinjection system (WPI PV830, Sarasota, FL, USA). The injected embryos were incubated in an incubator at 26 °C, and were transferred to a glass tank after hatching (10 dpf). F0 mutants were screened by restriction enzyme digestion and Sanger sequencing. Heterozygous mutants of *gata2a* and *gata2b* were generated by crossing the F0 *gata2a* or *gata2b* mutant males with normal females. Then, male and female fish with the same heterozygous *gata2a* or *gata2b* mutation were crossed to generate homozygous *gata2a* and *gata2b* mutants. Polyacrylamide gel electrophoresis (PAGE)-based heteroduplex mobility assays, restriction enzyme digestion and Sanger sequencing were performed to screen homozygous mutants. Three pairs (males and females) of heterozygous mutant fish of F1 generation were mated to produce F2 generation homozygous mutant fish. Each pair of the F1 generation can obtain about 150 F2 mutant fish at one mating, among which about 20 surviving homozygous mutant fish can be obtained. Each F1 pair should be mated at least three times. As for the *gata2a* F2 homozygous mutants, restriction endonuclease (*Hin*fI) were used for the genotyping instead of PAGE. As for the *gata2b* F2 homozygous mutants, there was no valid restriction endonuclease. In addition, PAGE electrophoresis is difficult to distinguish homozygous mutants from wild types due to small differences in base numbers (two base pairs). Therefore, PAGE can only identify heterozygous mutants, while homozygous mutants and wild types are identified by PCR product sequencing. The primers used in this study are listed in Supplementary Table S1.

### 4.3. Immunofluorescence

Nile tilapia larvae were collected at 5 (10 fish, 5 males and 5 females) and 30 dpf (6 fish, 3 males and 3 females), and eyes were isolated from Nile tilapia at 90 dpf (6 fish, 3 males and 3 females). The larvae and the eyes were subsequently fixed in 4% paraformaldehyde at 4 °C. Each sample was dehydrated, embedded in paraffin wax and sectioned at 5 μm. The sections were deparaffinized, hydrated and then stained with rabbit anti-Gata2a antibody (1:500; peptide antigen: KPGLHPAGSGYPCSSS) and then incubated with goat anti-rabbit Alexa Fluor Plus 594 (1:1000; Invitrogen, Carlsbad, CA, USA). Gata2a antibody was produced by a company (Abiotech, Jinan, China). Then, 4′,6′-Diamidine-2-phenylindole-dihydrochloride (DAPI) (Invitrogen, Carlsbad, CA, USA) was used for nuclear staining. Images were captured under a laser confocal microscope (Olympus FV3000, Tokyo, Japan).

### 4.4. Western Blot

Total protein was extracted from mutants and WT eyes at 60 dpf. The protein lysates were resolved by SDS/PAGE on 12% Tris·glycine gels followed by transfer to nitrocellulose membrane. Unspecific binding was blocked with 5% BSA in Tris-buffered saline with Tween-20 (TBST) for 1 h at room temperature. Incubation with Gata2a antibody at a dilution of 1:500 was performed overnight at 4 °C. After washing with TBST three times, the membrane was incubated with HRP-conjugated secondary antibody (Invitrogen, Carlsbad, CA, USA, 1:1000) in blocking solution for 1 h. The abundance of α-Tubulin was examined as a loading control using rabbit anti-α-Tubulin (Cell Signaling Technology, Beverly, MA, USA) at a dilution of 1:1000. Signal was detected with Pierce™ ECL Western Blotting Substrate (Invitrogen, Carlsbad, CA, USA) and was visualized on a Fusion FX7 (Vilber Lourmat, East Sussex, France).

### 4.5. Transcriptome Sequencing and Analysis

Eyes from at least three fish were separately harvested from WT and *gata2a* homozygous mutants at 35 dpf, and three biological replicates were performed. Total RNA was extracted from each sample using TRIzol reagent (Thermo Fisher Scientific, Waltham, MA, USA). Six libraries were constructed, including three WT fish libraries and three mutant libraries, and sequenced with Illumina NGS system (Personal Biotech, Nanjing, China). All clean reads were mapped to the tilapia genome sequence (https://www.ncbi.nlm.nih.gov/genome/?term=Niletilapia, 2 May 2017). The fragments per kilobase of exon per million fragments mapped (FPKM) method was used to calculate gene expression levels. The differentially expressed genes (DEGs) were classified according to the following criteria: genes meeting both “*p* value < 0.05” and “log2 (*gata2a*^−/−^_FPKM/WT_FPKM) > 1” statistical criteria were classified as up-regulated genes; in contrast, genes meeting both “*p* value < 0.05” and “log2 (*gata2a*^−/−^_FPKM/WT_FPKM) < −1” were classified as down-regulated genes. All the raw data were deposited in the NCBI Short Read Archive (accession number: PRJNA905808).

### 4.6. Histological Analysis

Larvae and eyes from WT (three fish for each age stage, one male and two females) and gata2a mutants (three fish for each age stage, one male and two females) were fixed in Bouin’s solution for 24 h at room temperature, and were then dehydrated and embedded in paraffin. The prepared samples were cross-sectioned at 5 μm, and each section was stained with hematoxylin and eosin. Images were captured with an Olympus BX51 light microscope (Olympus, Tokyo, Japan).

### 4.7. Real-Time PCR

Total RNAs were extracted from all samples using a column-based RNA extraction kit (Qiagen) specialized for small quantities of RNA. DNase I (RNase free) treatment and cDNA preparation were carried out according to the manufacturer’s instructions. Total RNA was directly used as a template for PCR as negative control to exclude the genomic DNA contamination. Real-time PCR was performed with Fast SYBR Green Master Mix (Takara) on a 7500Fast Real-Time PCR system (Applied Biosystems, Waltham, MA, USA). Primers were designed to target regions of the genome with *gata2a* and *gata2b* mutations. β-actin was used as the internal control. The relative abundance of target gene mRNA transcripts was evaluated using the formula: R = 2^−ΔΔCt^. At least three samples for each genotype were analyzed. Primer sequences used for real-time PCR are listed in Table of the Supplementary Data.

### 4.8. Data Analyses

All data were presented as the mean ± SD from at least three independent experiments. Statistical comparisons were made using Student’s *t*-test when comparing two groups. One-way ANOVA was performed for comparisons with more than two groups followed by Tukey’s test. Statistical analyses were performed using GraphPad Prism 8 (GraphPad Software, La Jolla, CA, USA). In all analyses, a value of *p* < 0.05 was considered to be statistically significant.

## Data Availability

The RNA-seq data used in this study have been deposited in the NCBI Short Read Archive under the accession number PRJNA905808.

## Supplementary Materials

The supporting information can be downloaded at: https://www.mdpi.com/article/10.3390/ijms24043567/s1.

## Abbreviations

*Dpf*	Day post fertilization
*Plf*	Primary lens fiber cells
*Slf*	Secondary lens fiber cells
*Ep*	epithelial cell
*PAM*	protospacer adjacent motif
*KEGG*	Kyoto Encyclopedia of Genes and Genomes
*DEGs*	differentially expressed genes
*FDR*	false discovery rate
*DAPI*	4′,6′-diamidine-2-phenylindole-dihydrochloride
*WT*	wild type
