# Supplementary material for: Gata2a Mutation Causes Progressive Microphthalmia and Blindness in Nile Tilapia
Source: Int J Mol Sci. 2023 Feb 10;24(4):3567. doi: 10.3390/ijms24043567 (PMC9958714; doi:10.3390/ijms24043567)
Supplement: Supplementary file 1 [file ijms-24-03567-s001.zip › ijms-2140967-supplementary.pdf]

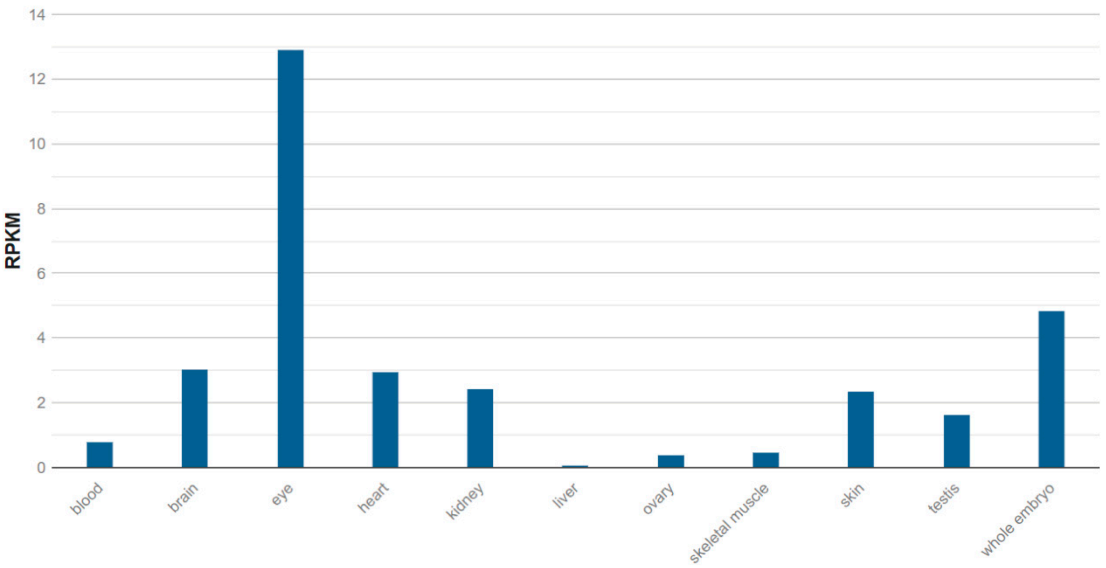

Figure S1 Expression pattern of *gata2a* in Nile tilapia transcriptomes from multiple tissues in the NCBI database (BioProject: PRJNA78915)

Gata2a is widely expressed in different tissues, with the highest expression in the eyes.

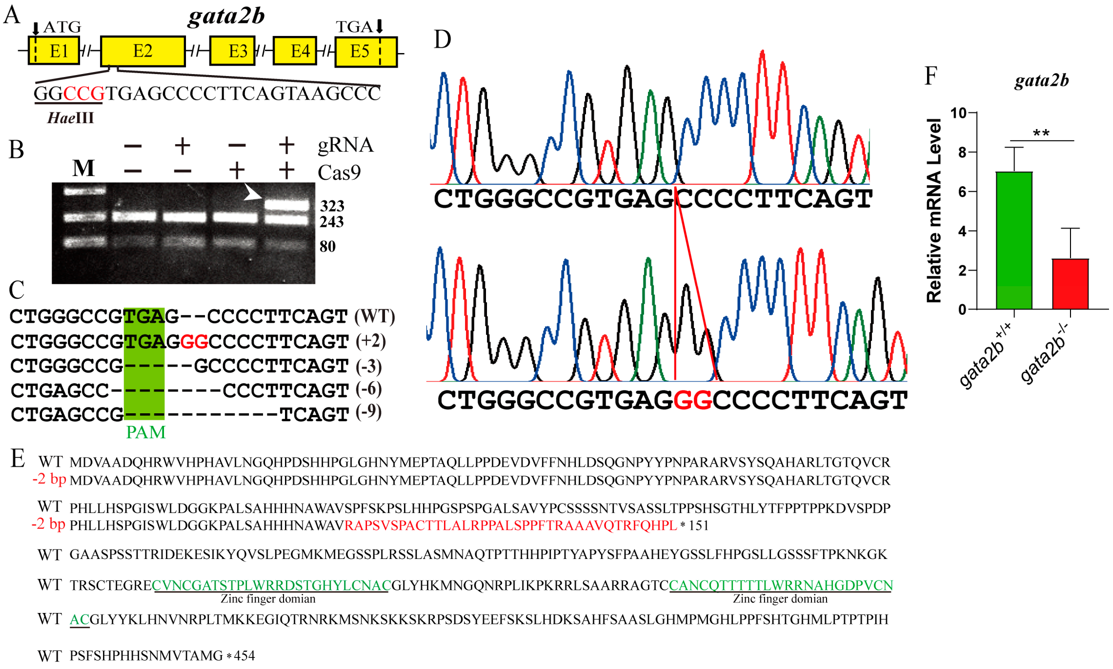

Figure S2 Generation of tilapia *gata2b* mutant line by CRISPR/Cas9

(A) Schematic diagram showing *gata2b* gene structure and sgRNA target site near the translation start site on the antisense strand. (B-F) Generation of the *gata2b* mutant line. Mutations at the *gata2b* locus were analyzed by restriction enzyme digestion (B) and DNA sequencing (C and D), and this deletion resulted in premature translation termination (E). The *gata2b* mRNA level was significantly down-regulated in the mutant (F). Deletions are indicated by dashes. The numbers in brackets represent the bases deleted/inserted from each allele. the frameshift-altered amino acid sequence for the mutated *gata2b* is indicated in red. M, marker; PAM, protospacer adjacent motif.

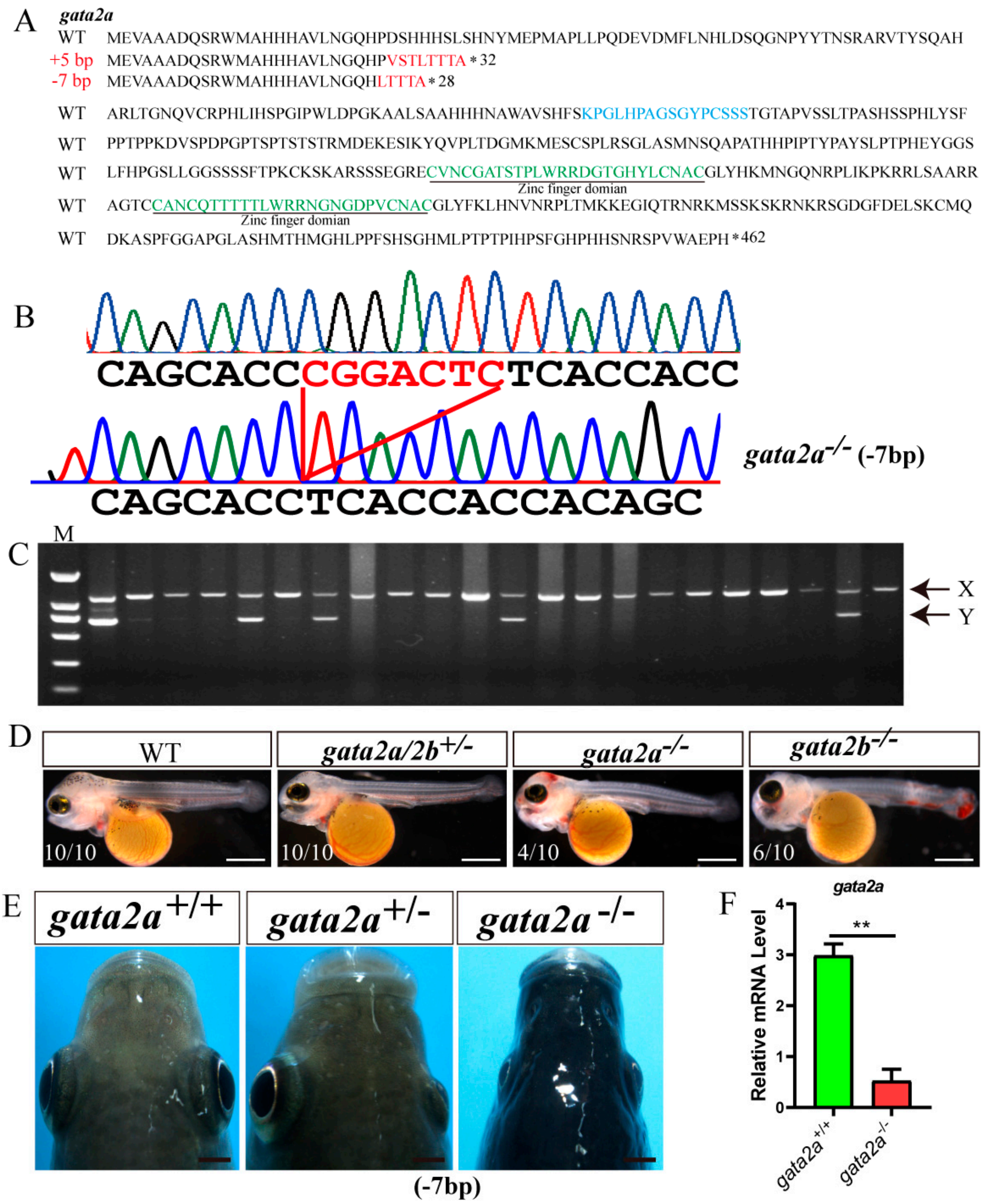

Figure S3 Homozygous mutants of *gata2a* with 7-bp deletion

(A) Amino acid sequences of WT and frameshift-altered *gata2a* mutants. (B) Sequencing results of *gata2a* gene from WT and homozygous mutants (-7 bp). (C) Example of sex genotyping for the F2 generation. (D) Mutants with hematopoiesis defects. About half of the *gata2a* (4/10) and *gata2b* (6/10) homozygous mutants with severe hematopoiesis defects during hatching and died after hatching. (E) Eyes morphology of the *gata2a* mutants (-7 bp). (F) The *gata2a* mRNA level was significantly down-regulated in the mutant. WT: wild type. M: marker.

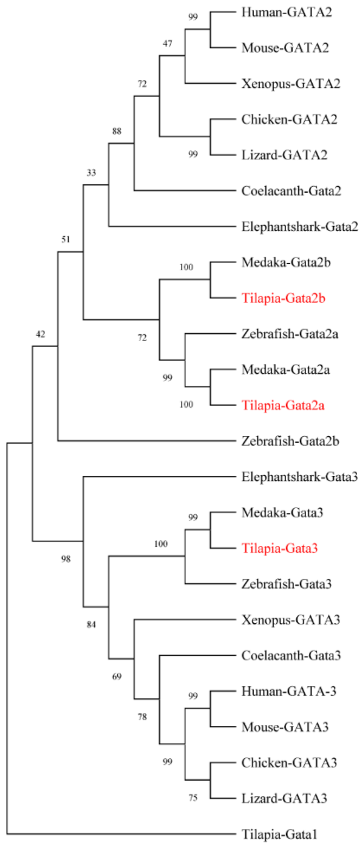

Figure S4 Phylogenetic analyses of vertebrate GATA2 and GATA3 gene

Phylogenetic tree constructed using MEGA11 with the Neighbor-Joining Algorithm method.

Table S1 Primers used in present study

Primer	Sequence （5'-3'）	Purpose
gata2a-gRNA-F1	TAATACGACTCACTATAGGCTGTGGTGGTGAGAGTCCGTTTTAGAGCTAGAAATAGC	CRISPR/Cas9
gata2b-gRNA-F1	TAATACGACTCACTATAGGGCTTACTGAAGGGGCTCAGTTTTAGAGCTAGAAATAGC	
gRNA-R1	AGCACCGACTCGGTGCCAC	
gata2a-T-F1	GACTTCCACTTGGAGGCAAA	Mutants screening
gata2a-T-R1	GGTTCAGAAACATATCAACC	
gata2b-T-F1	CCTCATCTCCTTCACAGCCC	
gata2b-T-R1	TCCTTCTCGTCGATTCTGGTAG	
gata2b-page-F1	GGATTTCGTGGCTGGACG	PAGE for *gata2b*
gata2b-page-R1	ACTGCTGCTGCACGGGTAA	
F5	ATGGCTCCGAGACCTTGACTG	Sex genotyping
R3	CAGAAATGTAGACGCCCAGGTAT	
gata2a-rt-F	AGAGAGACTGTCAGCTCA	Quantification of *gata2a* and *gata2b*
gata2a-rt-R	GTGCTGACCGGGTGCTGA	
gata2b-rt-F	TCTGACGGGGACTCAGGTCT	
gata2b-rt-R	TTACTGAAGGGGCCCTCA	
β-actin-rt-F	CATCCCGTCCTGCTCACA	Internal control
β-actin-Q-R	AGGCGTACAGGGACAGCA	
